# Seed Germination-Influencing Bioactive Secondary Metabolites Secreted by the Endophyte *Cladosporium cladosporioides* LWL5

**DOI:** 10.3390/molecules181215519

**Published:** 2013-12-13

**Authors:** Muhammad Waqas, Abdul Latif Khan, Liaqat Ali, Sang-Mo Kang, Yoon-Ha Kim, In-Jung Lee

**Affiliations:** 1 School of Applied Biosciences, College of Agriculture and Life Sciences, Kyungpook National University, Daegu 702-701, Korea; E-Mails: agronomist89@gmail.com (M.W.); latifepm78@yahoo.co.uk (A.L.K.); kmoya@daum.net (S.-M.K.); kimyoonha7979@gmail.com (Y.-H.K.); 2 Department of Agriculture Extension, Government of Khyber Pakhtunkhwa, Bunir 19290, Pakistan; 3 Department of Biological Sciences and Chemistry, College of Arts & Sciences, University of Nizwa, Nizwa 33, Oman; E-Mail: malikhejric@unizwa.edu.om (L.A.); 4 International Agricultural Training Center, Kyungpook National University, Daegu 702-701, Korea

**Keywords:** natural products, endophytes, lettuce seed, growth inhibition, seed germination, weeds control, allelopathy

## Abstract

The present study was aimed to isolate bioactive metabolites produced by a fungal endophyte from *Helianthus annuus*, *Capsicum annuum,* and *Cucumis sativus* and to assess their role in seed germination*.* Culture filtrate of the endophyte HA-3B from *H. annuus* was significantly inhibitory towards the germination and growth of lettuce seeds. HA-3B was identified as *Cladosporium cladosporioides* LWL5 through molecular techniques. Different concentrations (100, 500 and 1000 ppm) of the ethyl acetate extract obtained from the culture inhibited the lettuce seed germination. The extract was subjected to column chromatography and a bioassay-guided isolation method, which yielded compounds **1**, **2** and an oily fraction. The oily fraction, subjected to fractionation and spectroscopic techniques, resulted in the identification of 31 different constituents. Compounds **1** and **2** were identified and characterized through MS and NMR spectroscopic techniques as benzoic acid. The bioassay results showed that this compound significantly inhibited the growth and germination of lettuce seeds. In conclusion, assessing the role of endophytes harboring essential crop plants can help us to develop potentially eco-friendly herbicides.

## 1. Introduction

The increase in human population has overwhelmed the global output of food production. To tackle the global demand for food supply, an enormous amount of pressure has been created on agricultural resources. To obtain higher yields per unit area in a sustainable way has led to the extensive utilization of crop growth-regulating synthetic chemicals. These chemicals include herbicides, pesticides, fertilizers, and plant growth regulators [1]. The extensive use of synthetic chemicals has proved however unfriendly to the environment [2]. Besides the pollution of soil and water, it has greatly influenced the resistance of weeds to herbicides [3,4].

Natural resources, including fungal endophytes, are currently being explored for bioactive substances including medicines and agrochemicals like plant growth-regulating substances and bio-herbicides [5]. Fungal endophytes reside in the intercellular spaces of all organs of plants including the roots, stems, leaves and seeds without causing any disease symptoms. Plants offer protection to the endophytes while the endophytes secrete essential metabolites and increase plant’s fitness against various harsh environmental challenges [6,7]. Such chemical constituents can be growth-promoting or growth-inhibiting substances towards other living organisms [8,9]. Exploration of such bioactive metabolites can be allelopathic and may be used to inhibit the growth of neighboring competitive plants, specially the unwanted weeds in an agricultural system [9].

Looking at this potential, it was hypothesized that the capacity of plants to inhibit the growth of neighboring plants is due to specific metabolites produced by the plant or microbial symbionts associated with the plant’s root. Therefore, we selected three different crop plants well known for their allelopathic effects [10], *i.e.*, *Helianthus annuus*, *Capsicum annuum*, and *Cucumis sativus*, with the aim of isolating novel endophytes to investigate their allelopathic effect by identifying potent inhibitory metabolites through bioassay-guided isolation and spectroscopic identification techniques. For this purpose, lettuce seeds were taken as indicator species for differentiating growth-promoting or inhibiting effects.

## 2. Results and Discussion

The isolation of endophytic fungi from the roots of *Helianthus annuus*, *Capsicum annuum,* and *Cucumis sativus* yielded nine strains which were grouped on the basis of morphological trait similarity like colony shape, height and color of aerial hyphae, base color, surface texture and depth of growth into medium [11]. These strains were further cultured in Czapek broth media. The resultant culture filtrate (CF, 200 µL diluted to 5 mL in distilled water) was then applied to lettuce seeds. Control was treated with the same amount of distilled water. The effect on lettuce seed germination was observed after 72 h of incubation. Initial results revealed that the strain HA-3B isolated from *Helianthus annuus,* significantly inhibited lettuce seed germination and growth, followed by HA-2A as compared to control and other strains (Supplementary Information Figure S1). The strains CMS-5D and CMS-3B showed higher growth-promoting effects as compared to other strains, but none of them showed any promoting effect as compared to control. On the basis of the significant growth-inhibitory results, HA-3B was selected to further study its effects. The selected strain HA-3B was cultured in 4 L of Czapek broth media.

**Figure 1:**
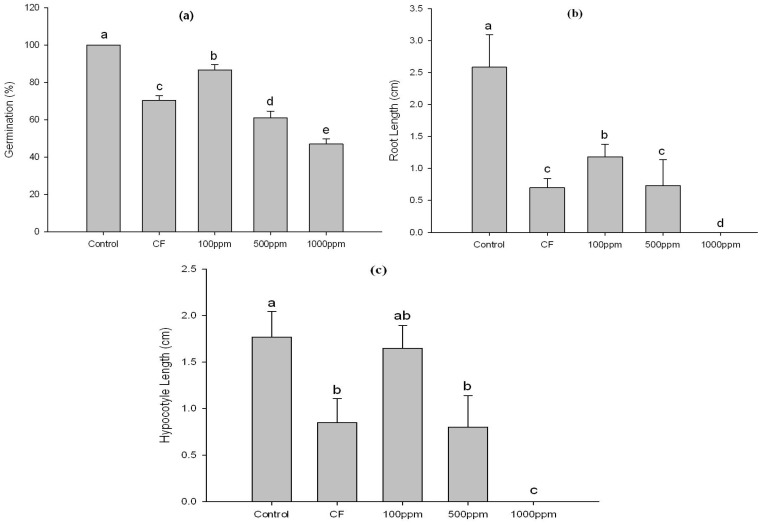
Assessment of *Cladosporium cladosporioides* LWL5 culture filtrate (CF) and its ethyl acetate extract (100, 500, 1000 ppm) on lettuce seed germination (**a**) root length (**b**) and hypocotyl length; and (**c**) Control was treated with distilled water. Different letters on bars indicates significant difference at (*p <* 0.05) among treatments as evaluated by Duncan’s Multiple Range Test (DMRT). Error bars represents the standard deviation (*n* = 45).

**Figure 2:**
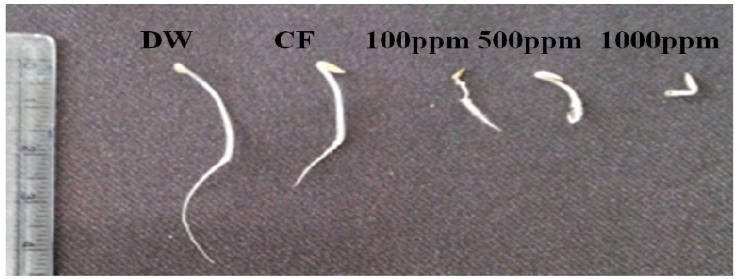
Inhibitory action of ethyl acetate extract obtained from CF of *Cladosporium cladosporioides* LWL5 at different concentration (100, 500 and 1000 ppm) on lettuce seed germination. Distilled water (DW) and culture filtrate (CF) were applied as a positive and negative control, respectively.

After incubation, CF was harvested through centrifugation and successively partitioned with ethyl acetate. A lettuce seed germination bioassay was performed with the resultant ethyl acetate extract at a concentration of 100, 500 and 1,000 ppm. In addition, CF of HA-3B and distilled water were applied again as a positive and negative control, respectively. After 72 h of incubation, the effects were assessed (Figure 1 and Figure 2). Based on results, the effect of CF and application of HA-3B ethyl acetate extract at 500 and 1000 ppm was inhibitory (*p* < 0.05) on lettuce germination. The germination rates of CF, and ethyl acetate extract (500 and 1000 ppm) were 70%, 61% and 47%, respectively, as compared to negative control (100%). Application of ethyl acetate extract at 1,000 ppm was 100% inhibitory (*p* < 0.05) of root and hypocotyl length (Figure 1). Culture filtrate and ethyl acetate extract at 500 ppm showed a similar effect and reduced the root and hypocotyl lengths by 71.7% and 54.8%, respectively.

Fungal mycelia obtained was used for DNA extraction, sequencing and phylogenetic analysis. The D1/D2 sequence of 28S rDNA regions was used for phylogenetic analysis with the help of MEGA software (5.0 version). The homologues sequence with lowest E values were BLASTn searched in NCBI. Using 1K bootstrap statistical support, a neighbor joining tree was constructed. The phylogenetic tree revealed 88% sequence homology with *Cladosporium* sp. and identified as a strain of *Cladosporium cladosporioides* LWL5 (Supplementary Information Figure S2).

**Figure 3:**
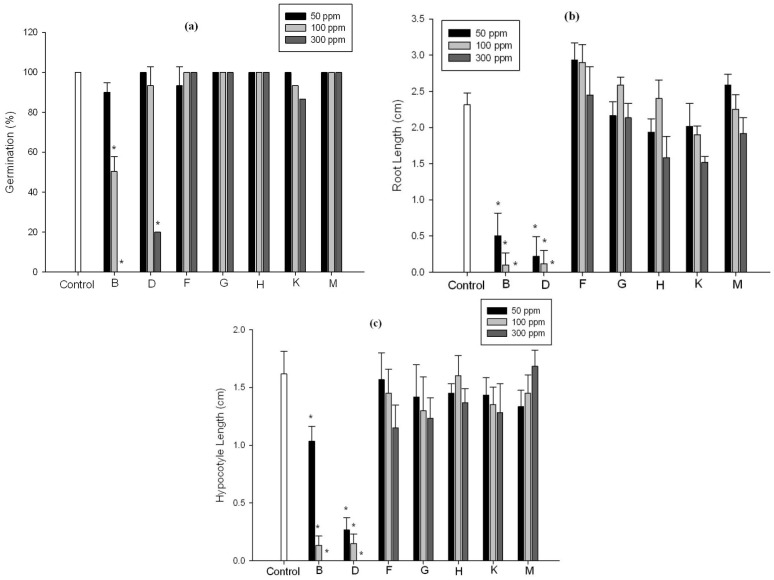
Effect of selected fractions on growth parameters of lettuce seeds. The effect was assessed at 50, 100 and 300 ppm on germination percentage (**a**), root length (**b**) and hypocotyl length (**c**). Star on bars indicates the significant difference at (*p <* 0.01) among treatments as analyzed by Duncan’s Multiple Range Test (DMRT). Error bars represents the standard deviation (*n* = 45). Fractions B, D, F, G, H, K and M were obtained after column chromatography from ethyl acetate extract of *C. cladosporioides* LWL5 culture filtrate.

**Figure 4:**
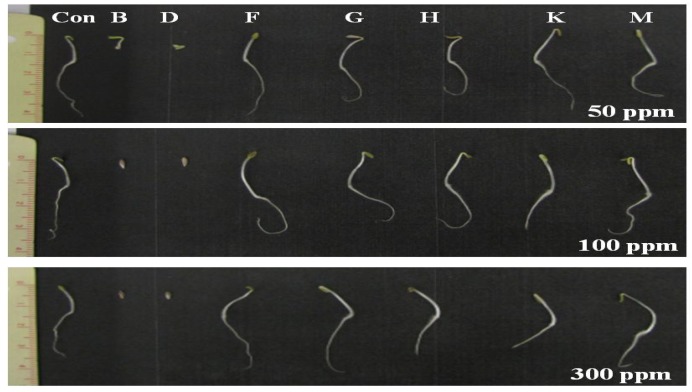
The effect of fractions B, D, F, G, H, K and M on lettuce seed germination and growth is compared more clearly at different concentrations. These fractions were obtained during column chromatography from ethyl acetate extract of *Cladosporium cladosporioides* LWL5 culture filtrate.

To find out the specific chemical constituent(s) responsible for growth inhibition, the extract was fractioned using column chromatography techniques. Based on TLC results (Supplementary Information Figure S3a), seven fractions were selected to assess their effects on the germination of lettuce seed. Various concentrations of these sub-fractions showed a varying effect on the growth of lettuce seeds. According to the results, fractions B (100 and 300 ppm) and D (300 ppm) significantly (*p <* 0.01) inhibited the germination of lettuce seeds by 57%, 100% and 80%, respectively, as compared to control and other sub-fractions (Figure 3 and Figure 4; Supplementary Information Figure S3b). In the case of root length, fractions B and D significantly (*p <* 0.01) reduced the length at 50, 100 and 300 ppm by 90%, 95% and 100%, respectively. Both fractions B and D displayed the same pattern of inhibitory effect on hypocotyl length.

Thin-layer chromatography (Supplementary Information Figure S3a) showed that fractions B and D were pure enough to proceed to identification and characterization through NMR and MS techniques. The molecular masses and corresponding molecular formulae were determined by the MS analysis, which indicated the presence of a molecular ion peak M^+^ at *m/z* 122, along with major fragments at *m/z* 105 (M^+^–OH) and 77 (M^+^–COOH). The structure of benzoic acid (BA) was further confirmed by the ^1^H- and ^13^C-NMR spectra. The ^1^H-NMR spectrum exhibited the characteristic signals for the mono-substituted benzene ring in the region δ 7.38–7.82, which was supported by three sp^2^ methine signals at δ 133.1 (C-2 and C-6), 116.2 (C-3 and C-5), and 129.6 (C-4). The quaternary carbon (C-1) appeared at δ 130.9, whereas the signal for carbonyl carbon (C-7) appeared at δ 163.5. All these spectroscopic observations and the comparison of the literature data proved the structure of benzoic acid [12,13]. 

Sub-fraction A, when vacuum dried yielded an oily sub-fraction (23.8 mg). Due to the immiscible nature of this fraction, the germination bioassay was performed using a dish pack method. The results showed that the lettuce seed germination was not affected by the application of oily sub-fraction. The sub-fraction was subjected to GC MS-MS SIM analysis to assess the presence of benzoic acid (Table 1). However, benzoic acid was not revealed, which suggests lack of bioactivity of the oily sub-fraction as compared to compound B and D.

**Table 1:** GC MS-MS analysis showing the chemical constituents of oily sub-fraction A.

Ser No.	Constituents	Retention Time (min)	Peak Area	% of Total
1	2-Tetradecane	20.292	16,058,256	1.199
2	Benzene, (1-butylhexyl)	22.875	15,474,933	1.156
3	Benzene, (1-propylheptyl)	23.051	15,536,730	1.160
4	Benzene, (1-ethyloctyl)	23.395	21,277,245	1.589
5	1-Hexadecene	23.880	13,660,024	1.020
6	Hexadecane	24.003	11,070,545	0.827
7	Benzene, (1-methylnonyl)	24.047	35,109,561	2.622
8	Benzene, (1-pentylhexyl)	24.497	31,801,835	2.375
9	Benzene, (1-butylheptyl)	24.559	62,881,071	4.696
10	Benzene, (1-propyloctyl)	24.744	62,173,747	4.643
11	Benzene, (1-ethylnonyl)	25.114	65,748,205	4.910
12	Heptadecane	25.643	11,864,656	0.886
13	Benzene, (1-methyldecyl)	25.740	100,035,495	7.470
14	Benzene, (1-pentylheptyl)	26.084	568,737,765	4.247
15	Benzene, (1-butyloctyl)	26.154	56,593,602	4.226
16	Benzene, (1-propylnonyl)	26.366	56,921,824	4.251
17	Benzene, (1-ethyldecyl)	26.727	68,110,143	5.086
18	Octadecane	27.203	18,626,915	1.391
19	Benzene, (1-methylundecyl)	27.344	92,660,685	6.920
20	Benzene, (1-pentyloctyl)	27.600	62,787,354	4.689
21	Benzene, (1- butylnonyl)	27.697	44,224,500	3.303
22	Benzene, (1- propyldecyl)	27.900	40,632,426	3.034
23	Benzene, (1-ethylundecyl)	28.270	54,728,342	4.087
24	Nonadecane	28.676	10,816,015	0.808
25	Benzene, (1-methyldodecyl)	28.861	63,294,054	4.727
26	Hexadecanoic acid, methyl ester	29.028	31,134,074	2.325
27	1,2-Benzenedicarboxylic acid	29.478	17,195,370	1.284
28	Eicosane	30.086	9,421,248	0.704
29	Octadecanoic acid, methyl ester	31.770	14,752,502	1.102
30	1,2-Benzenedicarboxylic acid, mono	36.653	137,557,985	10.272
31	7,8,17,18-Tetrahydro-35-methoxy-1, 3,21,23-tetramethyl-16H	51.137	40,081,884	2.993

It is well known that benzoic acid and its different derivatives reduce or inhibit seed germination and growth in various plants like *Betula platy phylla* var. *japonica* [12], *Pennisetum americanum* [14], and *Arabidopsis thaliana* [15]. BA induces disruption in cellular membrane integrity by lowering sulfhydryl group contents in cells, which ultimately causes lipid peroxidation [16]. Alteration in mineral uptake, chlorophyll contents, photosynthesis function, carbohydrate distribution, and hormonal actions are the consequences of lipid peroxidation due to BA [17]. Studies have elaborated the role of BA in seed growth as well. The effects of various carboxylic acids, including BA and its derivatives, have been reported as allelochemicals [12]. However, previously the source of BA in these experiments was either synthetic or different types of plant-derived allelochemicals. In the present study, BA was isolated from endophyte fungi *C. cladosporioides. C. cladosporioides* has been isolated as an endophytic fungus from different plant species like *Aconitum leucostomum* [18], *Astragalus adsurgens* Pall [19], and the marine sponge *Cliona* sp. [20]. Endophytes are known for their ability to produce similar kinds of natural products, which are isolated from the host plants [18]. Sunflower has been widely reported to be a source of various derivatives of BA [21,22] whilst the same was found in the culture of its associated endophyte in the present study. This further proves that endophytes harbored by host-plants can also produce similar kinds of constituents. This is in conformity to the findings of San-Martín * et al.* [20] and Krohn *et al.* [23]. In their study, they isolated *p*-methylbenzoic acid from *Cladosporium cladosporioides* and highly substituted benzoic acid derivatives from *Scytalidium* sp., respectively. The oily sub-fraction A was subjected to GC/MS-MS analysis which showed the absence of BA, however it showed other allelopathic compounds such as 1,2-benzenedicarboxylic acid. Ignacimutha [13] and Shanab *et al.* [24] isolated this compound previously. In conclusion, the present study indicates the potential role of crop plants’ associated endophytes. Such bioactive compound-producing endophytes can help to enhance the competition of host plants by producing allelochemicals such as BA, whilst suppressing competitors like weeds.

## 3. Experimental

### 3.1. General

The ^1^H- and ^13^C-NMR spectra were recorded in methanol-*d*_4_ using TMS as internal standard on an AVANCE III 500 spectrometer (Bruker, Madison, WI, USA) operating at 500 MHz. The chemical shift values are reported in ppm (δ) units and coupling constants (*J*) in Hz. For GC/MS an Agilent 7890A-5975C system with MSD (Agilent Technologies, Palo Alto, CA, USA) equipped with a JMS-HX-110 data acquisition system and JMS-DA 500 mass spectrometer was used. Methanol, ethyl acetate, hexane and Milli-Q water were used as elution solvents in chromatography.

### 3.2. Collection of Plant Samples for Isolation of Endophytic Fungi

Healthy *Helianthus annuus*, *Capsicum annuum,* and *Cucumis sativus* plants were carefully collected from the part of Kyungpook National University (Daegu, South Korea) fields with thick weed infestations and no weeding history during the growing season of the crop. This particular collection was made on the basis of the hypothesis laid out for this experiment and also earlier stated by Strobel *et al.* [5] that the chances of isolation of novel endophytes and bioactive compounds would be better in plants surviving in unique environmental settings. The roots were cautiously separated from the main stems of their respective plants and put in zip-locked plastic bags. The bags were immediately stored at 4 °C till the isolation process. The isolation process was carried under sterile conditions to minimize the chance of any contamination. Briefly, the roots were surface treated for sterilization with 2.5% sodium hypochlorite (30 min in shaking incubator at 120 rpm) and repeatedly washed with autoclaved distilled water (DDW) to remove any epiphytic microbes. Pieces of sterilized roots about 5 mm in length were plated onto the Hagem media (0.5% glucose, 0.05% KH_2_PO_4_, 0.05% MgSO_4_·7H_2_O, 0.05% NH_4_Cl, 0.1% FeCl_3_, 80 ppm streptomycin and 1.5% agar; pH 5.6 ± 0.2) [25]. In addition, imprints of plated roots were also taken on separate culture medium to ensure the effectiveness of surface sterilization [26]. The isolation process described by Waqas *et al*. was followed [26]. The newly emerged fungal spots were further grown and isolated on potato dextrose agar (PDA) medium. During isolation the endophytic fungal strains were repeatedly purified by the single hyphal tip method. The growing edge of mycelia having well separated hypha observed under light microscope were cultured on PDA medium to obtain pure cultures for each strain [27].

### 3.3. Screening of New Fungal Endophytes

In isolation, total nine different fungal strains were obtained and grown on PDA media. These strains were further grown in Czapek broth (50 mL; 1% glucose, 1% peptone, 0.05% KCl, 0.05% MgSO_4_·7H_2_O, and 0.001% FeSO_4_·7H_2_O; pH 7.3 ± 0.2) for seven days (shaking incubator 120 rpm; temperature 30 °C) [28]. The liquid culture and fungal mycelia were separated by centrifugation (2,500 ×*g* at 4 °C for 15 min). Pure culture filtrate (CF, 50 mL) and mycelium of each fungus were freeze-dried (Virtis Freeze Dryer, Gardiner, NY, USA) for 4–7 days. The lyophilized CF was diluted with 1 mL of autoclaved DDW, while the mycelia were used for genomic DNA extraction. For identification of growth inhibitory endophytes, bioassay was performed on *Lactuca sativa*, (LS) seeds. Fifteen seeds per replication and three replications per treatment were maintained. The seeds were grown on sterilized filter paper (70 mm ø, Type Roshi Kaisha, Ltd., Tokyo, Japan) in Petri dishes for 72 h (dark condition, 30 °C) inside incubator (HB-103, Hanbaek Scientific Co., Kyunggi-do, South Korea). The experiment was independently repeated five times. To each replication of treatment 5 mL of autoclaved DDW containing 200 μL of 1 mL diluted culture filtrate from media of all fungi was applied. Control was treated with autoclaved double distilled water. The procedure and instruments for further seed germination bioassay was kept the same, except for whenever the concentration needed to be changed.

### 3.4. Genomic DNA Extraction and Molecular Identification

On the basis of seed germination bioassay, HA-3B was selected for further investigation. The freeze dry mycelium was used for genomic DNA extraction according to [26]. The new isolate was identified by amplifying the 18S region of rDNA molecule by using universal primers: ITS-1; 5'-TCC GTA GGT GAA CCT GCG G-3' and ITS-4; 5'-TCC TCC GCT TAT TGA TAT GC-3' and large subunit (LSU; 28S rDNA) regions using primers LR0R (F) (5'-ACC CGC TGA ACT TA AGC-3') [29] and TW13 (R) (5'-GGT CCG TGT TTC AAG ACG-3'). Phylogenetic analysis was performed using the neighbor joining method. A dendrogram was constructed after nucleotide sequence similarity of the ITS region through the BLASTn search program and were aligned through CLUSTAL W using MEGA version 5.0. Bootstrap replications (1K) were used as a statistical support for the nodes in the phylogenetic tree.

### 3.5. Culture Conditions of the Selected Endophyte for Further Investigation

Based on the initial results and desired growth inhibiting activity, HA-3B was selected for further investigation. The Czapek culture broth containing conidia of the HA-3B were transferred to 4 L of Czapek broth. The broth was kept for 30 days in shaking incubator (30 °C at 120 rpm). After incubation, the supernatant and fungal mycelia were separated through centrifugation (at 5,000 ×*g* at 4 °C for 15 min).

### 3.6. Chromatographic Procedure for Bioassay-Guided Isolation and Extraction of Compounds

The supernatant obtained after centrifugation was subjected to bioassay-guided isolation. The supernatant was further treated with an equal volume of ethyl acetate (EtOAc) three times to obtain an extract. Upon extraction a crude hard gummy extract (3.6 g) was obtained. The EtOAc extract of the CF was once again bioassayed on LS seeds before chromatography. Upon detection of significant inhibitory effects at 100, 500 and 1000 ppm concentration the extract was further subjected to column chromatography. Silica gel (high purity grade, Davisil Grade 633, pore size 60 Å, 60–100 mesh, Sigma Aldrich, St. Louis, MO, USA) was used as a stationary phase. A slurry in eluent was prepared from the gummy extract and poured over the column with care to prevent bubble formation. A total of 13 eluents system (5, 10, 20, 40, 80% EtOAc in hexane, 100% EtOAc, 2, 4, 8, 20 and 40% methanol in EtOAc, 100% MeOH, and 2% water in MeOH) was run through the column. Finally, seven different fractions were initially collected from all eluent systems on the basis of thin layer chromatography (TLC). TLC was carried out on pre-coated Kieselgel 60 F254 (0.25 mm, Merck, Darmstadt, Germany). Spots were visualized with phosphomolybdic acid solution, followed by heating and observation under UV light of 254 nm and 365 nm wavelength (Vilber Lourmat, Cedex 1, France). These fractions were dissolved in DDW and bioassayed on LC seeds as described earlier to sort out the bioactive ones. The structures of compounds identified on the basis of bioactivity and TLC analysis were elucidated through NMR and GC/MS techniques.

### 3.7. Characterization of Benzoic Acid

The structure of benzoic acid was elucidated on the basis of bioactivity and TLC analysis and then verified through NMR and GC/MS techniques. MS: *m/z* 122 (M^+^), 105 (M^+^–OH), 77 (M^+^–COOH); ^1^H-NMR: δ 7.82 (2H, dd, *J* = 8.4 Hz, C-2 & C-6), 7.66 (1H, t, *J* = 7.7 Hz), 7.38 (2H, dd, *J* = 8.4 Hz, C-3 & C-5); ^13^C-NMR: δ 163.5 (C-7), 133.1 (C-2 & C-6), 130.9 (C-1), 129.6 (C-4), 116.2 (C-3 & C-5).

### 3.8. GC MS-MS Analysis of the Oily Sub-Fraction

In the present study, column chromatography of the ethyl acetate extract also resulted in an oily sub- fraction A. This was analyzed by GC MS MS on a HP-5MS column (30 m × 0.25 mm (i.d.), 0.25 µm film thickness). Ten mg of the oily sub-fraction was dissolved in 2 mL HPLC grade CH_2_Cl_2_ and 2 µL was injected in a split ratio of 1:20 for analysis with an ionization energy of 70 eV. Helium carrier gas was maintained at a head pressure of 30 kPa. The oven was programmed with a starting temperature of 60 °C and a final one of 285 °C. Oil constituents were identified by comparison of their retention indices and mass spectra with those of the online NIST MS spectra search program.

### 3.9. Statistical Analysis

The initial screening germination bioassay was independently repeated five times under the same experimental conditions. During the initial screening germination bioassay, CF of all isolated endophytes were applied on lettuce seeds and visual observation was considered enough for sorting out the growth inhibiting endophytes showing consistent effects. The data from the effect of ethyl acetate extract and its various fractions obtained after column chromatography on lettuce seed germination bioassay (repeated three times) was analyzed by one-way ANOVA compatible with a completely randomized design. Onward, Duncan’s Multiple Range Tests (DMRT) at *p* < 0.05 and *p* < 0.01 using Statistic Analysis System (SAS 9.1, San Diego, CA, USA) were applied to identify the significant differences among the mean values of various treatments [28,30].

## 4. Conclusions

Endophytes are well known for their natural products. To seek possible growth inhibitory compounds, bioassay-guided isolation in combination with column chromatography and mass spectrometry were used. The endophyte *Cladosporium cladosporioides* LWL5 isolated from *Helianthus annuus* produces metabolites and showed potent inhibitory activity. Benzoic acid was identified for the first time as the responsible growth inhibitory metabolite from *Cladosporium cladosporioides* LWL5. It is assumed that such endophytes producing specific metabolites can be the possible reason for mechanisms behind the unique attributes of host plants for which they are known. Moreover, the use of such endophytes can be extended for better weed control in sustainable agriculture.

## Supplementary Materials

Supplementary materials can be accessed at: http://www.mdpi.com/1420-3049/18/12/15519/s1.
