# Supplementary material for: Seed Germination-Influencing Bioactive Secondary Metabolites Secreted by the Endophyte Cladosporium cladosporioides LWL5
Source: Molecules. 2013 Dec 13;18(12):15519–30. doi: 10.3390/molecules181215519 (PMC6269724; doi:10.3390/molecules181215519)

**Supplementary Information**

**Figure S1.** Initial assessment of culture filtrate (CF) of fungal endophytes for their growth inhibitory/promotory effect on lettuce seed germination. The fungal endophytes strains were isolated from the roots of *Helianthus annuus*, *Capsicum annuum,* and *Cucumis sativus* grown in Czapek media. The CF were harvested through centrifugation, and bioassay was independently performed five times (*n* = 45). For convenient access the strains were given names starting with HA, CAA, CMS, referring to the strain-host plant *Helianthus annuus*, *Capsicum annuum,* or *Cucumis sativus*, respectively, followed by their isolation sequence number.

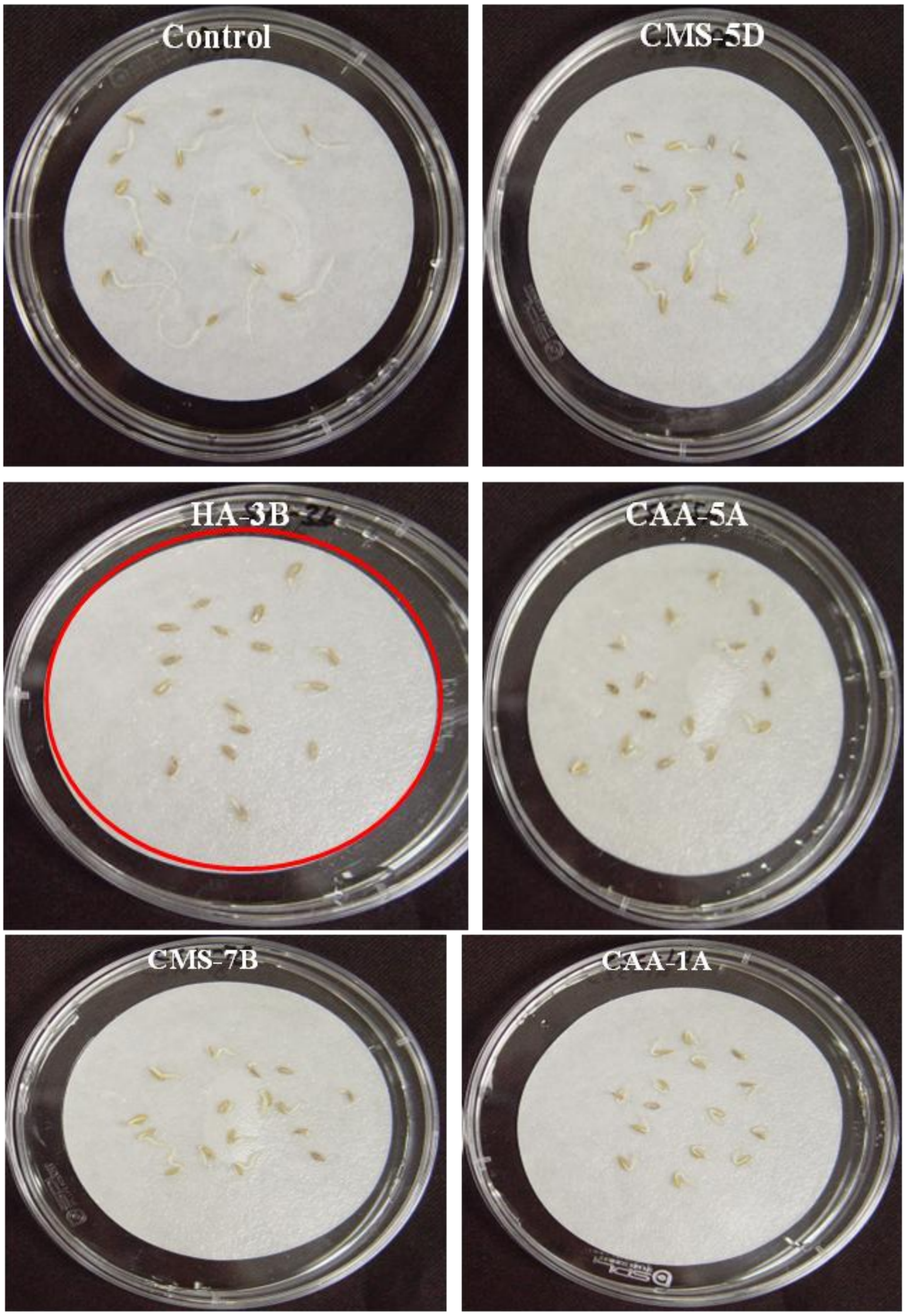

**Figure S1.** *Cont.*

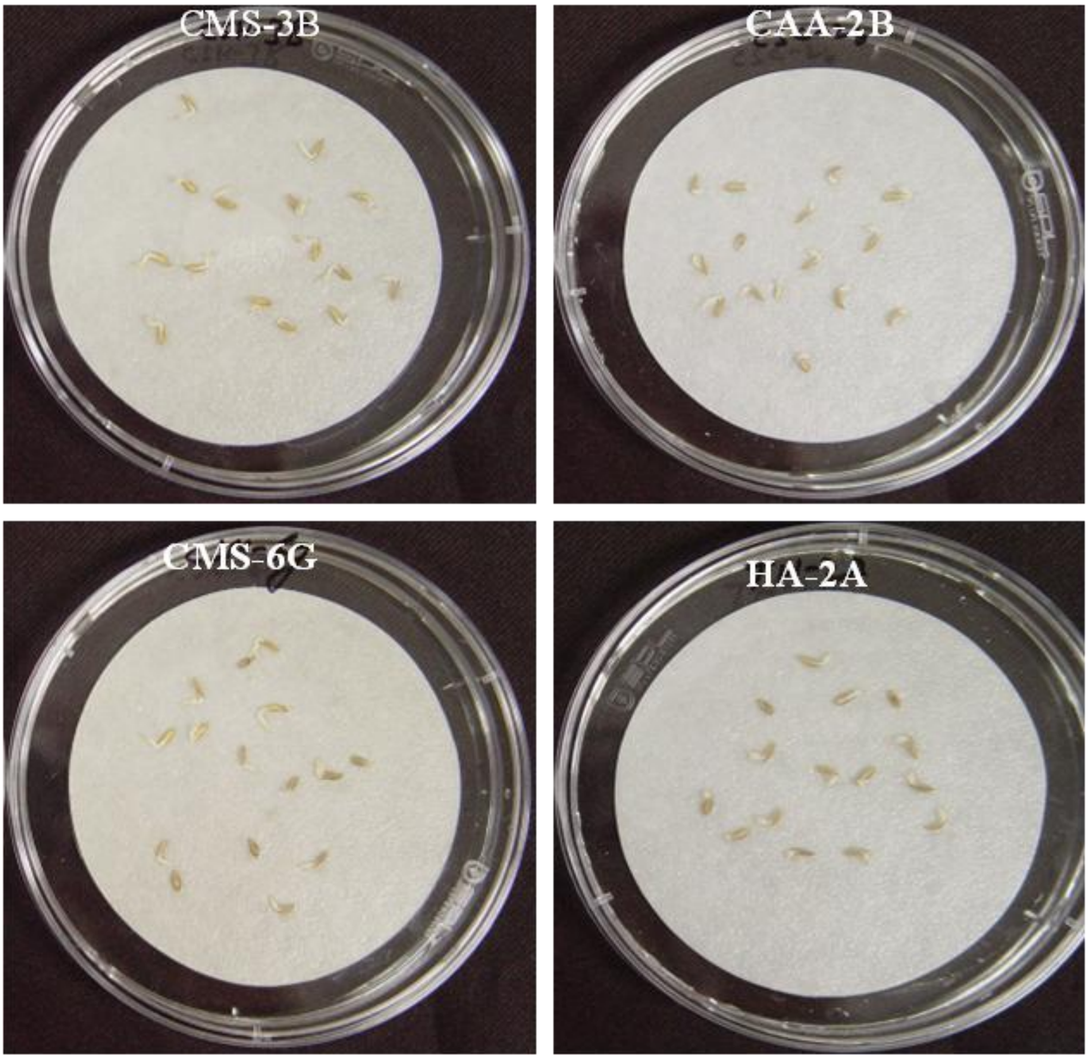

**Figure S2.** Phylogenetic analysis of strain HA-3B, isolated from the roots of sunflower. Phylogenetic tree was constructed by neighbor joining method of 22 clades (21 references and one clone) using MEGA software (version 5). The fungal strain HA-3B was identified as *Cladosporium cladosporiods* LWL5 as revealed by 88% bootstrap support of *C. cladosporiods.*

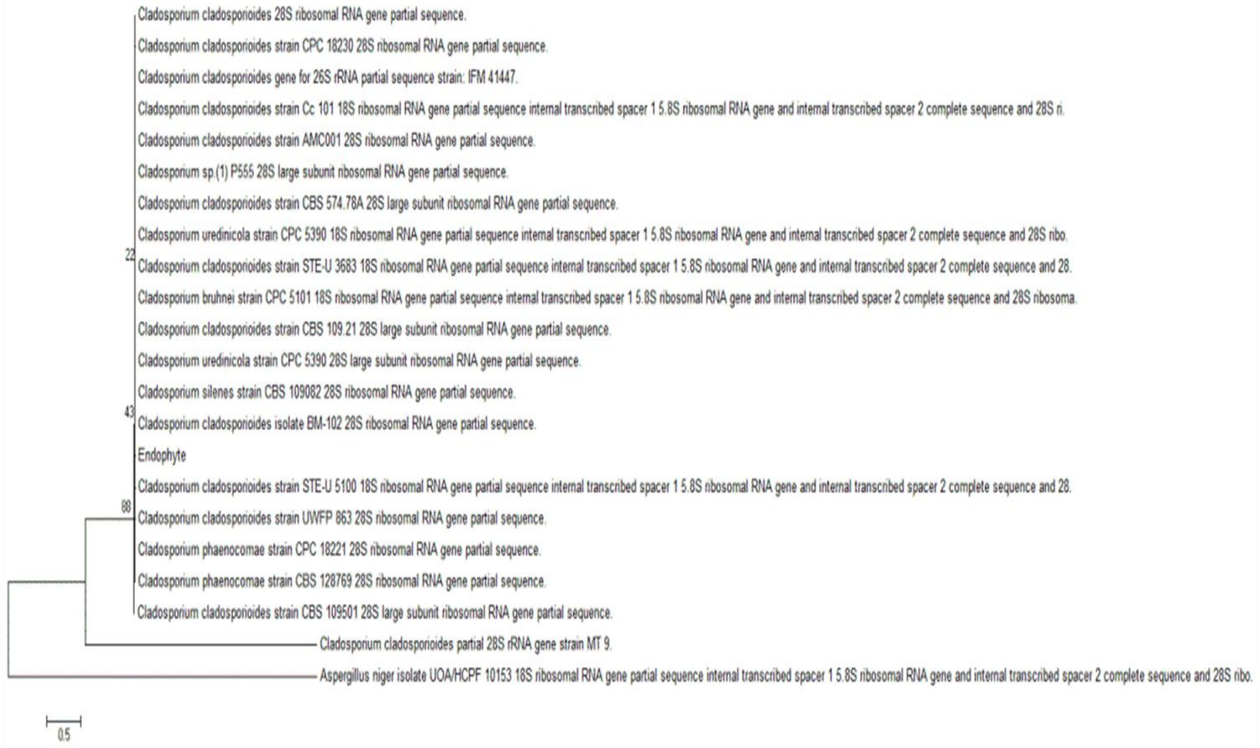

**Figure S3.** (**a**) TLC plates of fractions B, D, F, G, H, K and M visualized under 254 nm and 365 nm; (**b**) Effect of fraction B (elution with 10:80 ethyl acetate-hexane) and D (elution with 40:60 ethyl acetate–hexane) on lettuce seed germination and growth at 50, 100 and 300 ppm.

(a)

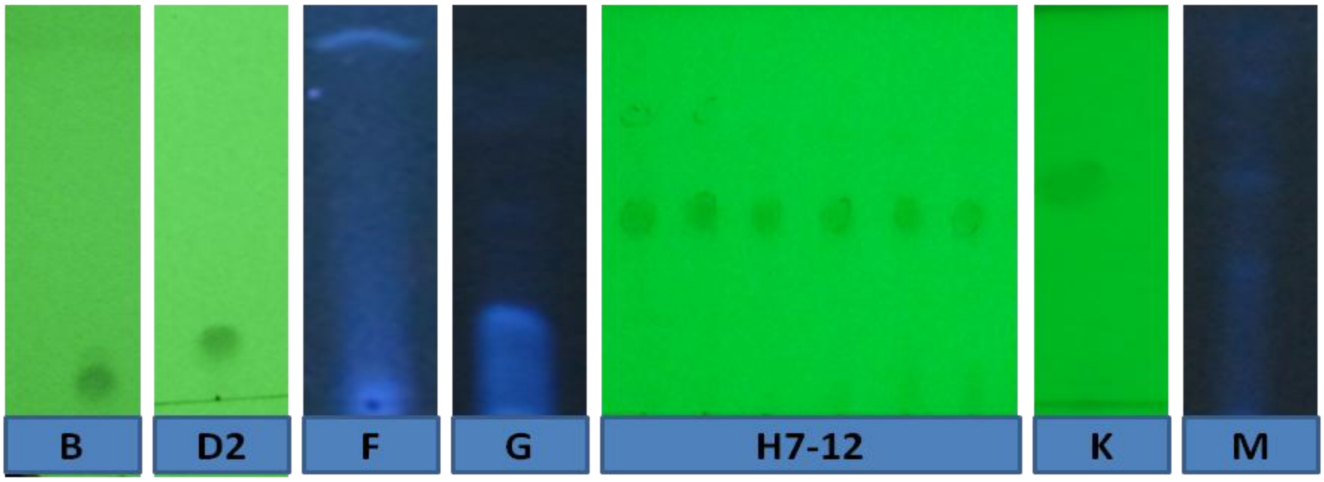


(b)

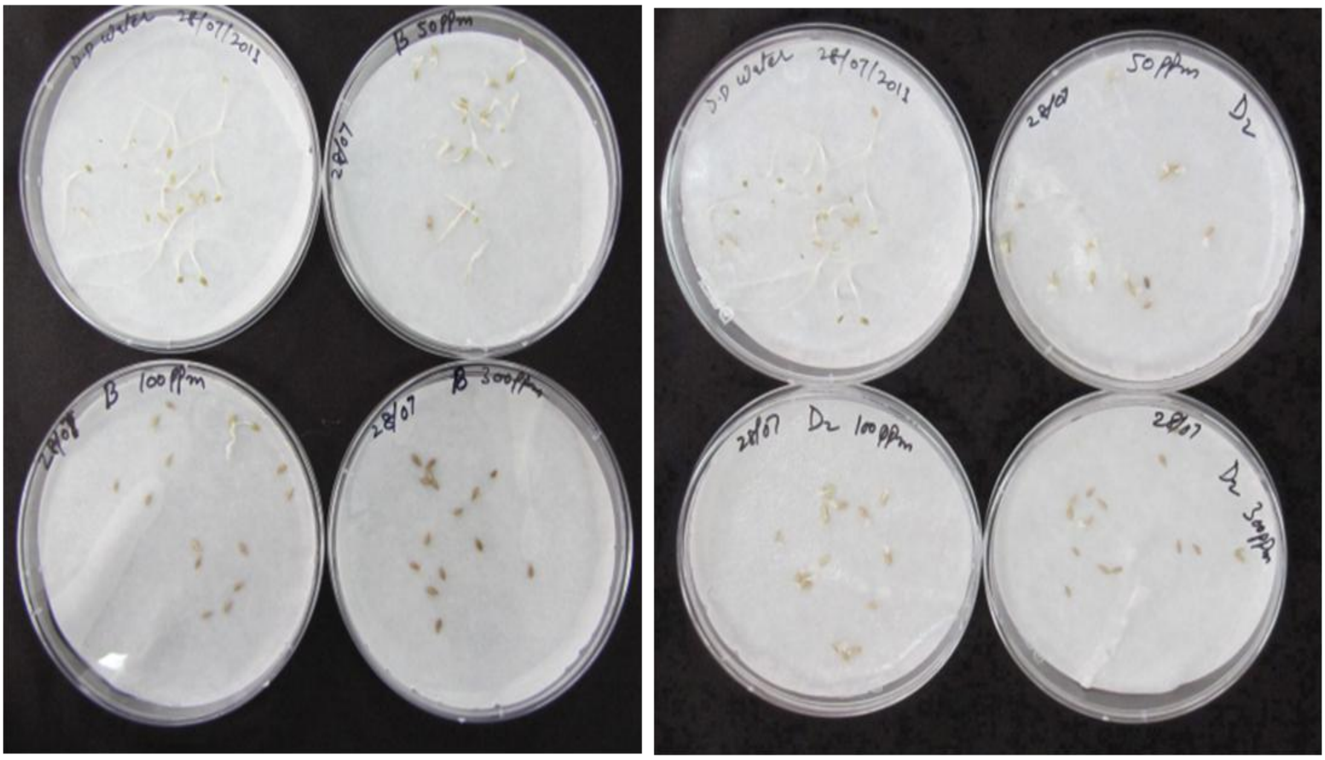

Supplement: Supplementary file 1 [file molecules-18-15519-s001.pdf]
